# Supplementary material for: Engineered Microvessel for Cell Culture in Simulated Microgravity
Source: Int J Mol Sci. 2021 Jun 13;22(12):6331. doi: 10.3390/ijms22126331 (PMC8231837; doi:10.3390/ijms22126331)
Supplement: Supplementary file 1 [file ijms-22-06331-s001.zip › ijms-1231159-supplementary.pdf]

Supplementary Material

Engineered Microvessel for Cell Culture in Simulated Microgravity

Mei ElGindi, Ibrahim Hamid Ibrahim, Jiranuwat Sapudom, Anna Garcia-Sabate and Jeremy C. M. Teo *

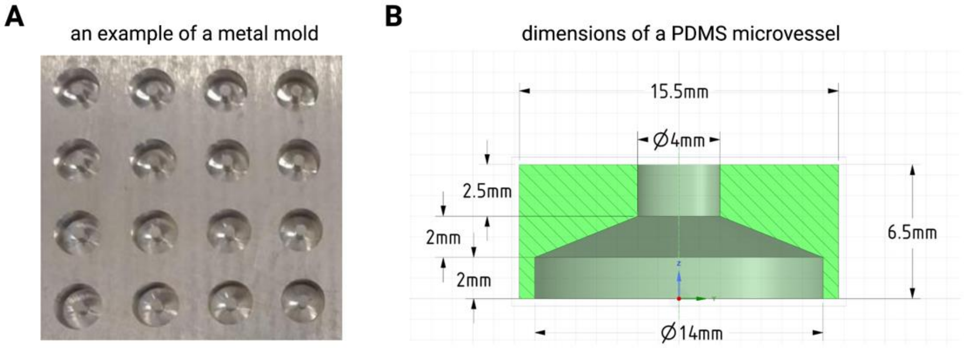

Figure S1. Dimensions of metal mold for PDMS microvessel casting. (**A**) Representative image of mold used for casting PDMS microvessels. (**B**) Dimensions of PDMS mold design.
